# Supplementary figures and images for: Effect of extracts from eggs of Helix aspersa maxima and Helix aspersa aspersa snails on Caco-2 colon cancer cells
Source: PeerJ. 2022 Apr 12;10:e13217. doi: 10.7717/peerj.13217 (PMC9012176; doi:10.7717/peerj.13217)

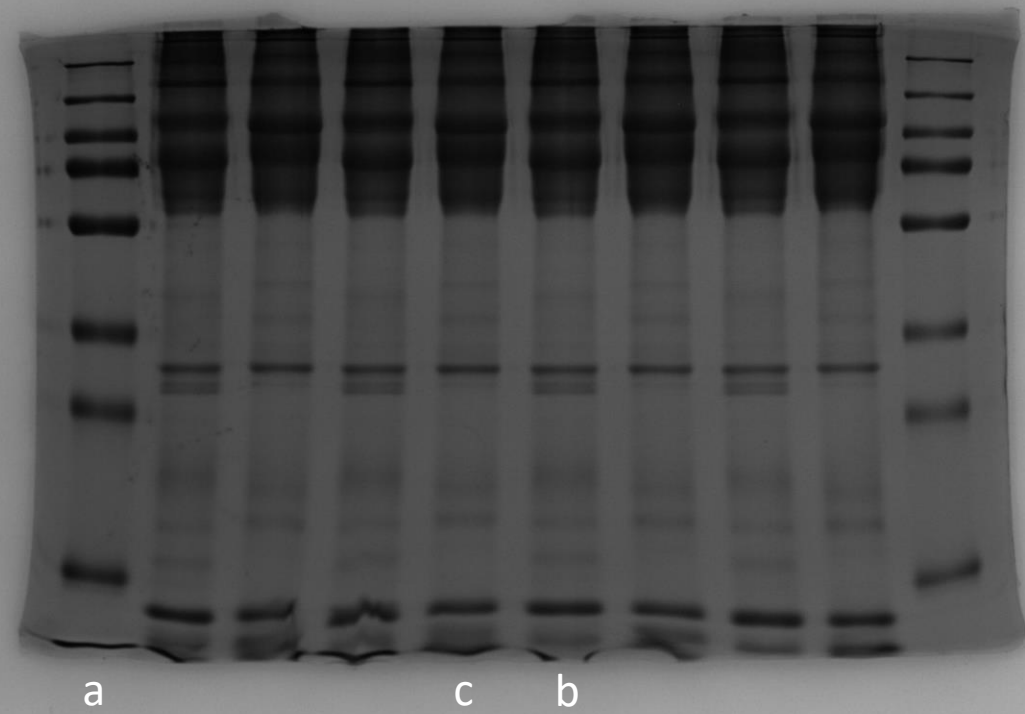

Figure 1A

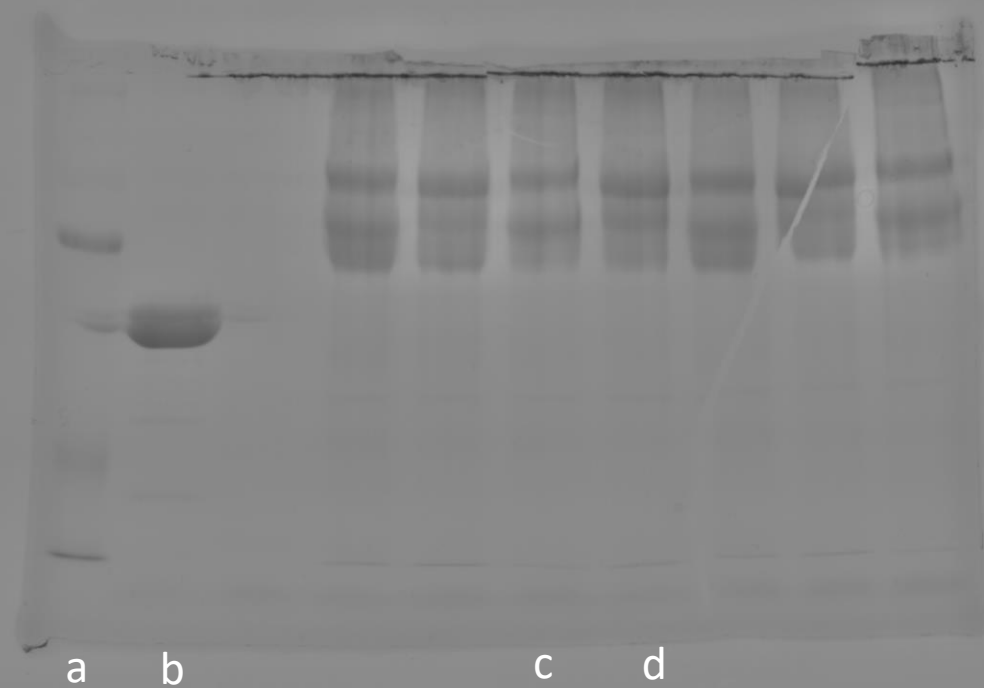

Figure 1B

Supplement: Supplemental Information 2 [file peerj-10-13217-s002.pdf]
